# Focus on POCUS

**DOI:** 10.31486/toj.26.0001

**Published:** 2026

**Authors:** Joseph Koveleskie, Bobby D. Nossaman

**Affiliations:** ^1^ Department of Anesthesiology and Perioperative Medicine, Ochsner Clinic Foundation, New Orleans, LA; ^2^ The University of Queensland Medical School, Ochsner Clinical School, New Orleans, LA

## TO THE EDITOR

Pulmonary aspiration during induction of general anesthesia remains one of the most feared perioperative complications, particularly in patients with small bowel obstruction because gastric distension and retained enteric contents may substantially increase regurgitation risk.^1-3^ Despite decades of clinical practice favoring nasogastric tube decompression in these patients, the evidence supporting routine preinduction placement remains surprisingly limited.

Historically, gastric decompression was adopted on sound physiologic grounds.^4^ Early surgical reports described transnasal drainage techniques that relieved vomiting, abdominal distension, and patient discomfort by evacuating accumulated gastric and intestinal contents.^4^ These observations continue to influence contemporary perioperative management. In addition, nasogastric tube placement may facilitate administration of water-soluble contrast agents used in modern small bowel obstruction treatment pathways.^5,6^

However, physiologic plausibility should not be mistaken for proven perioperative benefit. Most modern studies examining nasogastric tube use in patients with small bowel obstruction focused on nonoperative management outcomes rather than anesthesia-specific endpoints. Available observational studies and systematic reviews have not consistently demonstrated improvements in the need for surgery, bowel ischemia, or time to resolution with routine decompression.^7-9^ Importantly, no direct evidence demonstrates that routine preoperative nasogastric tube placement reduces aspiration during induction of anesthesia.^10^

Routine placement is also not benign. Complications may include mucosal trauma, malposition, electrolyte disturbances, and significant patient discomfort.^10^ In selected circumstances, an improperly functioning tube may provide false reassurance rather than meaningful protection.

Modern anesthetic practice offers additional safeguards, including head-elevated positioning, rapid-sequence induction, cuffed tracheal intubation, videolaryngoscopy, and point-of-care ultrasound (POCUS) to assess gastric contents.^2,3^ These tools support a more individualized strategy by identifying patients at greatest risk while avoiding unnecessary invasive intervention in others.

Accordingly, we suggest that routine preinduction nasogastric tube placement for all patients with small bowel obstruction should be reconsidered. A selective, risk-based approach, guided by clinical presentation, degree of distension, ongoing emesis, inability to tolerate supine positioning, and gastric ultrasound when available, appears more consistent with current evidence and modern airway management.^11^

Further prospective research focused specifically on peri-induction aspiration outcomes would help resolve this long-standing question.
